# Solitary Neurofibroma of the Floor of the Mouth: A Case Report

**DOI:** 10.1155/2011/967896

**Published:** 2011-11-20

**Authors:** Motoyoshi Maruyama, Hiroaki Fushiki, Yukio Watanabe

**Affiliations:** Department of Otolaryngology, Head & Neck Surgery, University of Toyama, 2630 Sugitani, Toyama-shi, Toyama 930-0194, Japan

## Abstract

We present a case of a solitary neurofibroma of the floor of the mouth protruding into the submandibular region. A 51-year-old female presented with a 2-year history of swelling of the floor of the mouth. MRI revealed that the mass measuring 50 × 70 mm showed a homogenous, low signal intensity on a T1-weighted image and high signal intensity on a T2-weighted image. The tumor was completely removed through a cervical approach. Cases of a solitary neurofibroma originating from the floor of the mouth are extremely rare.

## 1. Introduction

Neurofibroma is characterized as a benign, slow-growing neoplasm. It may appear as a solitary tumor or have multiple localizations, as in von Recklinghausen disease (VRD). When a neurofibroma grows in the head and neck, it presents with symptoms, such as upper airway obstruction, swallowing difficulty, mastication deficits, or cosmetic distortion of the face [1]. Neurofibroma arising from the floor of the mouth is extremely rare. In this paper, we describe an unusual case of neurofibroma of the floor of the mouth developing and protruding slowly into the submandibular region.

## 2. Case Report

**Figure 1:**
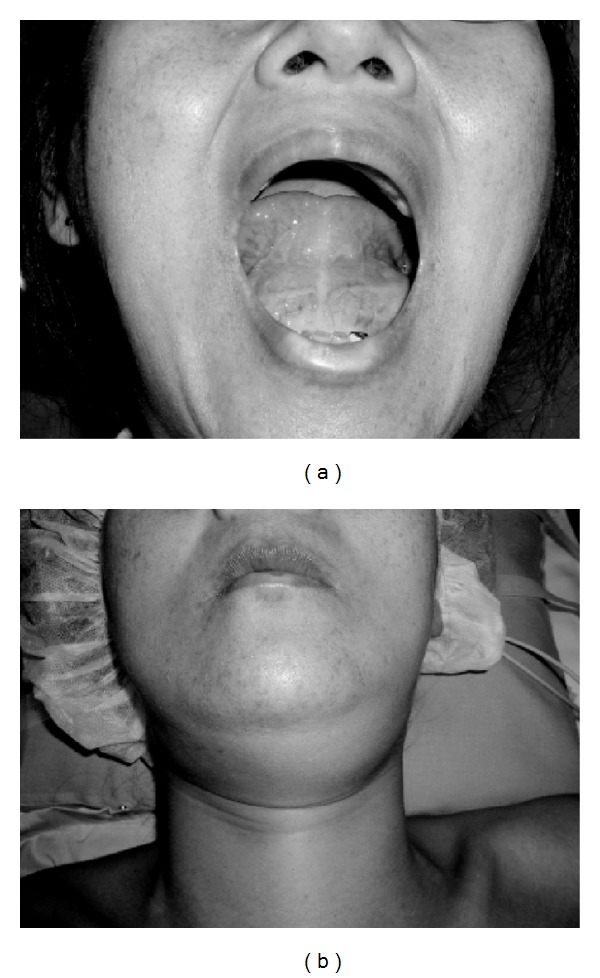
Clinical appearance of the floor of the mouth (a) and submandibular (b). The tongue was deviated to the upper side, and the submental and left submandibular regions were swollen.

**Figure 2:**
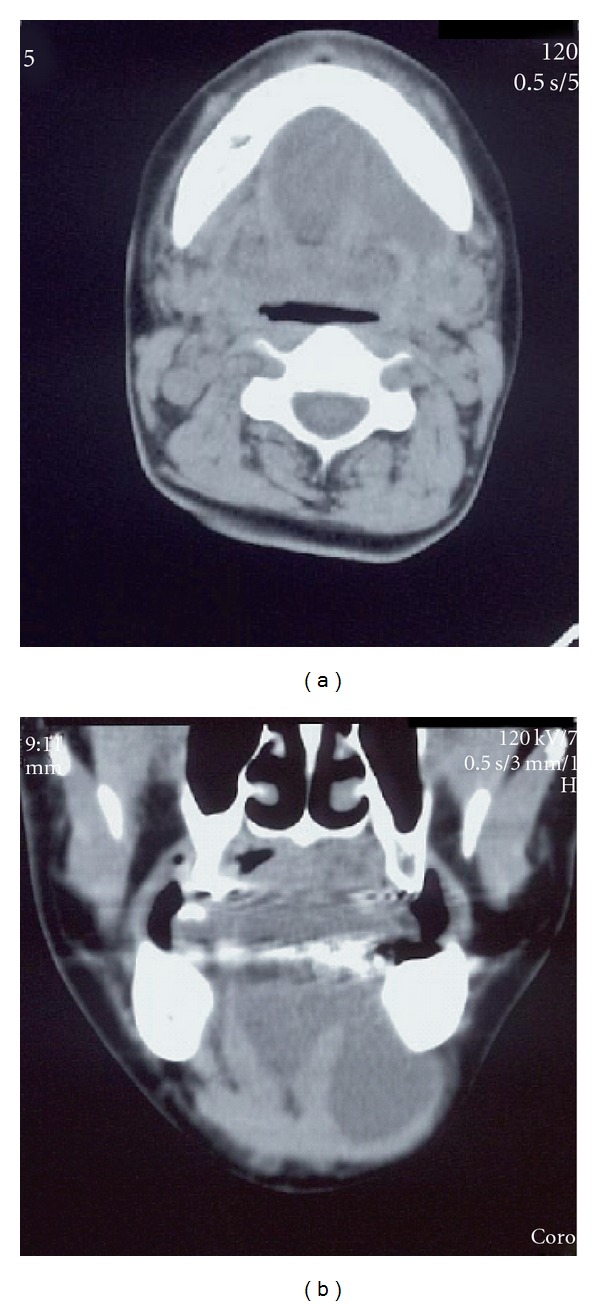
CT images. Axial (a) and coronal (b) images. CT revealing a mass of low density extending from the floor of the mouth to the left submandibular region.

**Figure 3:**
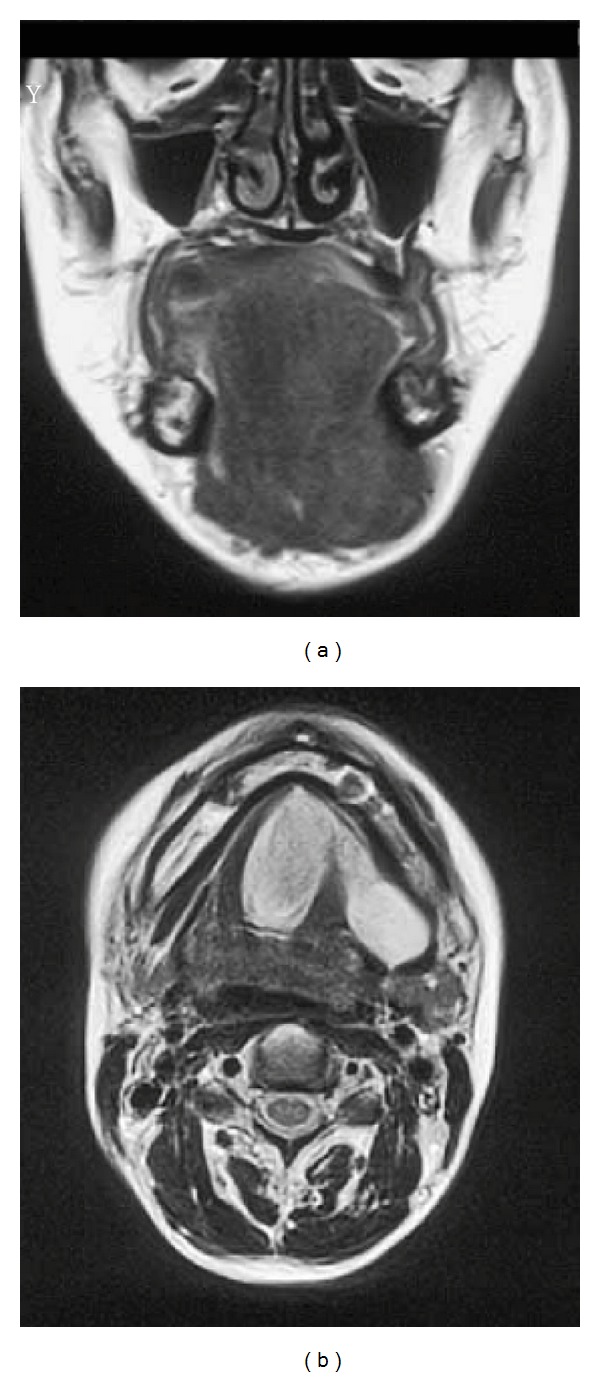
MRI images. Coronal T1-weighted image (a) and axial T2-weighted image (b). MRI revealed that the mass showed a homogenous low signal intensity on the T1-weighted image and high signal intensity on the T2-weighted image.

A 51-year-old female presented with a 2-year history of swelling of the floor of the mouth. She was a deaf-mute since 2 years of age. Physical examination revealed deviation of the tongue to the upper side and swelling of the submandibular regions (Figure 1). She was complaining of a swelling of the floor of the mouth but did not complain of dyspnea, swallowing difficulties, mastication, or phonation deficits. Blood tests were within normal limits, and a CT scan showed a low-density mass extending from the floor of the mouth to the mandible and protruding from the mylohyoid muscle (Figure 2). MRI revealed that the mass measuring 50 × 70 mm showed a homogenous, low signal intensity on a T1-weighted image and high signal intensity on a T2-weighted image (Figure 3). The tumor was completely removed through a cervical approach under general anesthesia. The postoperative course was uncomplicated, and the patient was discharged. The position of the tongue returned to normal. The patient presented with neither taste disturbances nor sensitivity disturbances.

**Figure 4:**
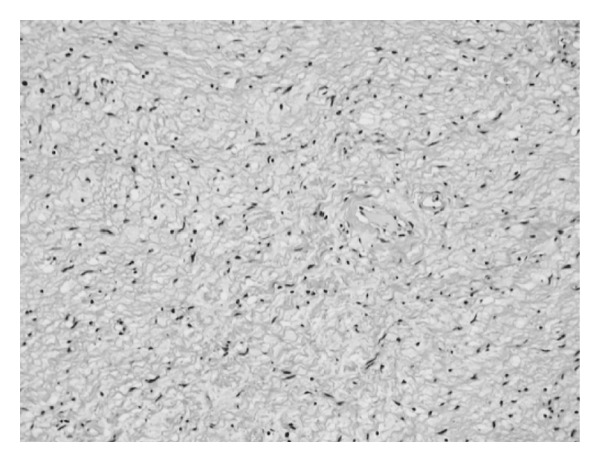
Histological findings. Interlacing bundles of spindle cells with hyperchromatic nuclei (hematoxylin and eosin, 100x).

 Microscopy revealed that the circumscribed tumor consisted of interlacing bundles of spindle cells with hyperchromatic nuclei. Spindle cells were intermixed with foci comprising dense bundles of collagen fibers. Pathological diagnosis was a neurofibroma with no signs of malignancy (Figure 4). Subsequent examination revealed no manifestations of VRD, and the patient's family history showed that no members of the family suffered from VRD. She had no café au lait spots and was followedup for 1 year with no signs of recurrence.

## 3. Discussion

Neurofibromas of the floor of the mouth are extremely rare. Among 66 neurofibromas in the facial region, the following distribution was found in the literature: tongue, 12; palate, 12; mandibular ridge/vestibule, 15; maxillary ridge/vestibule, 9; buccal mucosa, 10; lip, 4; mandibular intrabony, 2; gingiva, 1. Cases originating from the floor of the mouth were reported in the 1970s in the literature but not described in detail [2, 3]. This study reports a case of a solitary and relatively huge neurofibroma of the floor of the mouth protruding into the submandibular region. Papadopoulos et al. [4] presumed that intraoral neurofibromas may originate from the branches of the fifth and, occasionally, the seventh cranial nerve. In our case, the patient presented with neither taste disturbances nor sensitivity disturbances after surgery; hence, the cranial nerve branches from which the tumor may have arisen remain unclear. 

Generally, surgical excision has been the standard treatment for neurofibromas. In some cases, complete excision requires the sacrifice of cranial nerves, which subsequently causes significant functional deficits of the upper aerodigestive tract or substantial cosmetic deformity. Cases of surgical excision of floor-of-the-mouth lesions have not been reported to date, but the intraoral approach is a better treatment for lesions measuring less than 60 mm, such as a dermoid cyst that frequently occurs in the floor of the mouth [5]. Care should be taken not to damage the lingual nerve and Wharton's duct. Cervical incision is another option when the tumor is bigger and protrudes from the mylohyoid muscle layer although it leaves a visible scar on the neck. The risk of malignant transformation is significant, that is, between 3 and 15%, especially in VRD [6–8].

## References

[1] Greinwald J, Derkay CS, Schechter GL (1996). Management of massive head and neck neurofibromas in children. *The American Journal of Otolaryngology*.

[2] Chen SY, Miller AS (1979). Neurofibroma and schwannoma of the oral cavity. a clinical and ultrastructural study. *Oral Surgery Oral Medicine and Oral Pathology*.

[3] Cherrick HM, Eversole LR (1971). Benign neural sheath neoplasm of the oral cavity: report of thirty-seven cases. *Oral Surgery Oral Medicine and Oral Pathology*.

[4] Papadopoulos H, Zachariades N, Angelopoulos AP (1981). Neurofibroma of the mandible: review of the literature and report of a case. *International Journal of Oral Surgery*.

[5] Miyamaru S, Ogata N, Takemura T Two cases of Giant Cyst in the floor of the mouth. *Otolaryngology—Head and Neck Surgery*.

[6] Loutfy WG, Ryan DE, Toohill RJ, Meyer GA (1990). Trigeminal nerve neurofibroma: case report. *Journal of Oral and Maxillofacial Surgery*.

[7] Regezi JA, Sciubba J (1993). *Oral Pathology: Clinical-Pathologic Correlations*.

[8] Stewart A, Bailey BMW (1992). Neurofibroma of the inferior alveolar nerve: diagnostic and management difficulties. *The British Journal of Oral and Maxillofacial Surgery*.

